# 

**Published:** 1875-01

**Authors:** 


### CONTENTS VOL. XXIX.

### A

| | |
|---|---|
| Anæsthetics . . . . . . . . . . | 13 |
| Abnormal Calcareous Formation in the Human System | 101 |
| American Dental Society of Europe . . . | 143 |
| Address . . . . . . . . . . | 145 |
| Artificial Teeth on Protruding Gums . . . | 170 |
| Address . . . . . . . . . . | 173 |
| Alveolar Abscess—Treatment with Carvacrol . . | 201 |
| Anatomy . . . . . . . . . . | 246 |
| Alternation, a law of vital action . . . . | 277 |
| American Dental Association . . . . . | 319 |
| " " Convention . . . . . | 320 |
| A New Automatic Mallet . . . . . | 320 |
| Amalgam . . . . . . . . . . | 459 |
| A Common Cause of Apoplexy . . . . | 497 |
| Amalgam . . . . . . . . . . | 502 |
| American Dental Association . . . . . | 507 |
| " " Convention . . . . . | 508 |

Address of B. Oscar Doyle, D. D. S. . . . 524
Associations . . . . . . . . 549

### B

Bibliographical . . . . . . . . 94
" . . . . . . . 374
Biography . . . . . . . . 500

### C

Comments on the Recent Address of Dr. Allport, before the Academy of Dental Science, Boston, Mass . 28
Correspondence . . . . . . . 40
Cohesive and Non-Cohesive Gold . . . . 70
Commencement . . . . . . . 187, 93
Chloroform Condemned . . . . . 106
C. W. Spalding . . . . . . . 223
Celluloid . . . . . . . . . 315
Cases in Practice . . . . . . . 457
Celluloid—A new Apparatus . . . . . 463
Carbonic Oxide in Tobacco Smoke . . . 499
Celluloid . . . . . . . . . 547
Care of the Teeth during Gestation . . . 628

### D

Dental Pathology and Surgery . . . . . 142
Death from Chloroform . . . . . . 172
Dental Education . . . . . . . 207
Discussions before the Miss. Valley Dental Society . 212
Dental Pulp and Pulpless Teeth . . . . 242
Dental Education . , . . . . 465, 270, 252
Dental Society of the State of New York . . 272
Does past Experience justify the Continued Use of Cohesive Gold . . . . . . . . . 429
Dental Legislation . . . . . . . 509
Dentistry and Dental Physicians . . . . 527
Dental Vulcanite Company . . . . . 529
Dental Legislation . . . . . . . 597

### E

Eastern Indiana Dental Association . . . 231, 548
Exposed Pulps . . . . . . . . . 493
Ether and Chloroform as Anæsthetics . . . 496
Educational . . . . . . . . . 506
Early History of Practical Anatomy . . . 606

### H

How we Apples Swim . . . . . . 301

### I

Introductory Lecture, Ohio Dental College . . 23
Inverting the Body in Chloroform Poisoning . . 73
Injury of the Face . . . . . . . . 139
Illinois State Dental Society . . . . . . 232
Is Dentistry a Specialty of Medicine . . . 448
Iowa State Dental Society . . . . . . 446
Irregularity of the Teeth and Treatment . . . 483
Is the Profession of Dental Surgery in its relations to young men, holding its own with other professions and pursuits . . . . . . . . 489
Irregularities of the Teeth and the best means of correcting the same . . . . . . . . 515
In Memoria—Dr. A. Blake . . . . . . 550

### K

Kentucky State Dental Society . . . . 537, 222

### L

Loss of the Tongue . . . . . . . . 184
Local Peculiarities and Diseases of the Teeth in Europe 571

### M

Mechanical Appliances in Operative Dentistry . 34
Minutes of the Ohio State Dental Society . . 88
Miss. Valley Dental Society . . . . . . 93
Malleting . . . . . . . . . . 204
Miss. Valley Dental Association . . . . 263, 307

Mechanical *vs.* Operative Dentistry . . . . 426
Minutes and Discussions before Indiana Dental Asso. 452
Michigan University Dental College . . . 462
Merrimac Valley Dental Society . . . . 596
Michigan State Dental Society . . . . 596
Married . . . . . . . . . 632

N

New Gold . . . . . . . . . 143
Northern Ohio Dental Association . . . 272
Neuralgia . . . . . . . . 291
New Jersey State Dental Society . . . 320, 537
Nutrition . . . . . . . . . 562

O

Ohio Dental Society . . . . . . . 43
Obituary . . . . . . . . . 54
Ohio Dental College Association . . . 93, 188
On taking Plaster Impressions . . . . 136
Operative *vs.* Mechanical Dentistry . . . . 167
Ohio Dental College . . . . . . . 464
Ohio State Dental Society . . . . . 591, 596

P

Pathology of Decay . . . . . . . 76
Personal . . . . . . . . . 92
Prevention and Treatment of Approximate Decay . 160
Prohibition of Chloroform in Anæsthesia . . 421
Physiology . . . . . . . . . 440
Personal . . . . . . . . . 464
Professional Courtesy . . . . . . 471
Pathology and Treatment of Exposed Pulps . . 553

R

Review of Dr. Allports Proposition to separate the Mechanical from other departments of Dental Practice 57
Report of Discussions before the Ohio Dental Society . . . . . . . . 108, 88

Report on Dental Education . . . . . . 181

S

Southern Dental Association . . . . . . 52
S. S. White Dental Engine . . . . . . 144
Salicylic Acid . . . . . . . 233, 240, 270
Sensitive Dentine . . . . . . . . 463
State Board of Dental Examiners . . . . 592

T

Twenty-ninth Volume . . . . . . . 4
The Dental Pain Obtunder . . . . . . 99
The Same Old Story . . . . . . . 186
The Miss. Valley Dental Association . . . 188
Treatment of Exposed Pulps and Criticism of Discussions before the Ohio State Dental Society . . 312
Tenn. Dental Association . . . . 318, 271, 532
The Small Catechism . . . . . . . 317
Treatment of Chronic Alveolar Abscess . . . 436
The Dental Profession in England . . . . 474
The Diagnosis of the Lead Line . . . . 498
The Goal of Medical Science . . . . . 499
Treatment of Dead Teeth . . . . . . . 520
The List of Societies . . . . . . . . 547
The Extraction of Teeth . . . . . . . 620
The List of Dentists in Ohio . . . . . . 626
The Dental Law . . . . . . . . 627
The Close of the Volume . . . . . . 631

W

What about the Rubber Business now? . . . . 96
What Every Dentist Should Know . . . . 296

